# A Comparison of Petiole Hydraulics and Aquaporin Expression in an Anisohydric and Isohydric Cultivar of Grapevine in Response to Water-Stress Induced Cavitation

**DOI:** 10.3389/fpls.2017.01893

**Published:** 2017-11-07

**Authors:** Megan C. Shelden, Rebecca Vandeleur, Brent N. Kaiser, Stephen D. Tyerman

**Affiliations:** ^1^ARC Centre of Excellence in Plant Energy Biology, School of Agriculture, Food and Wine, University of Adelaide, Adelaide, SA, Australia; ^2^Centre for Carbon, Water and Food, School of Life and Environmental Sciences, Faculty of Science, University of Sydney, Sydney, NSW, Australia

**Keywords:** aquaporin, cavitation, water-stress, isohydric, anisohydric, petiole, hydraulic conductivity, xylem embolism

## Abstract

We report physiological, anatomical and molecular differences in two economically important grapevine (*Vitis vinifera* L.) cultivars cv. Grenache (near-isohydric) and Chardonnay (anisohydric) in their response to water-stress induced cavitation. The aim of the study was to compare organ vulnerability (petiole and stem) to cavitation by measuring ultrasonic acoustic emissions (UAE) and percent loss of conductance of potted grapevines subject to the onset of water-stress. Leaf (ψ_L_) and stem water potential (ψ_S_), stomatal conductance (*g*_s_), transpiration (*E*), petiole hydraulics (*K*_Pet_), and xylem diameter were also measured. Chardonnay displayed hydraulic segmentation based on UAE, with cavitation occurring at a less negative ψ_L_ in the petiole than in the stem. Vulnerability segmentation was not observed in Grenache, with both petioles and stems equally vulnerable to cavitation. Leaf water potential that induced 50% of maximum UAE was significantly different between petioles and stems in Chardonnay (ψ_50Petiole_ = -1.14 and ψ_50Stem_ = -2.24 MPa) but not in Grenache (ψ_50Petiole_ = -0.73 and ψ_50Stem_ = -0.78 MPa). Grenache stems appeared more susceptible to water-stress induced cavitation than Chardonnay stems. Grenache displayed (on average) a higher *K*_Pet_ likely due to the presence of larger xylem vessels. A close relationship between petiole hydraulic properties and vine water status was observed in Chardonnay but not in Grenache. Transcriptional analysis of aquaporins in the petioles and leaves (*VvPIP1;1, VvPIP2;1, VvPIP2;2 VvPIP2;3, VvTIP1;1*, and *VvTIP2;1*) showed differential regulation diurnally and in response to water-stress. *VvPIP2;1* showed strong diurnal regulation in the petioles and leaves of both cultivars with expression highest predawn. Expression of *VvPIP2;1* and *VvPIP2;2* responded to ψ_L_ and ψ_S_ in both cultivars indicating the expression of these two genes are closely linked to vine water status. Expression of several aquaporin genes correlated with gas exchange measurements, however, these genes differed between cultivars. In summary, the data shows two contrasting responses in petiole hydraulics and aquaporin expression between the near-isohydric cultivar, Grenache and anisohydric cultivar, Chardonnay.

## Introduction

Grapevines respond to water deficit with a variety of physiological and molecular mechanisms including modifications to the liquid pathways of water movement through the root and shoot, and vapor movement through stomata (Lovisolo et al., 2010). Under conditions of water-stress, grapevines are susceptible to xylem cavitation and embolism (Tyree and Sperry, 1989) resulting in reduced hydraulic conductivity of the xylem pathway. Cultivar and species differences have been observed in *Vitis* sp. in their physiological responses to drought stress (Schultz, 2003; Soar et al., 2006; Alsina et al., 2007; Vandeleur et al., 2009; Gerzon et al., 2015) indicating that variation in drought tolerance is a genetically controlled trait that can extend to cultivar differences.

Vulnerability to xylem cavitation is dependent on the hydraulic architecture of plants, a feature that varies between and within many plant species (Tyree et al., 1994; Schultz, 2003; Alsina et al., 2007). This variation is influenced by the segmented structure of dicotyledonous plants that permits hydraulic segmentation of different plant organs. In *Juglans regia* L. (Tyree et al., 1993), *Acer saccharinum* L. (Tsuda and Tyree, 1997), and *Vitis* sp. (Lovisolo et al., 2008a; Charrier et al., 2016; Hochberg et al., 2016), petioles have been shown to be vulnerable to cavitation under drought conditions, encouraging leaf shedding and stem preservation against further water-stress. Leaf shedding is also known to occur in grapevines under extreme episodes of drought (Keller, 2005).

The ability of plants to repair cavitated and embolized vessels is necessary to maintain the hydraulic pathway following drought (Zwieniecki and Holbrook, 2009), but how this occurs is not fully understood and refilling under negative pressure is still widely debated (Cochard et al., 2013, 2015). Holbrook et al. (2001) demonstrated using nuclear magnetic resonance (NMR) imaging that upon re-watering following drought treatment, grapevines were able to refill embolized vessels but only under non-transpiring conditions. Brodersen et al. (2010) subsequently demonstrated refilling of embolized vessels in *V. vinifera* (cv. Chardonnay) using high-resolution X-ray computed tomography. Flow rates from surrounding cells were quantified and successful refilling was demonstrated in stems that were under tension, but the repairing conduit were considered to be hydraulically isolated from the bulk of the xylem. It has been proposed that aquaporin’s located in the surrounding xylem parenchyma cells may contribute to water movement required for embolism recovery (Holbrook and Zwieniecki, 1999; Tyree et al., 1999; Lovisolo et al., 2010). More recently, models have been proposed describing how refilling of xylem conduits may occur through aquaporin facilitated movement of water via the phloem and living xylem parenchyma cells (Holbrook and Zwieniecki, 1999; Nardini et al., 2011; Brodersen and McElrone, 2013; Secchi et al., 2016).

In walnut (*J. regia*), increased expression of two aquaporin genes, *JrPIP2;1* and *JrPIP2;2* in parenchyma cells associated with xylem vessels, was found to correlate with refilling after winter embolism (Sakr et al., 2003). Kaldenhoff et al. (2008) reported that reduced expression of *NtPIP2* aquaporin (using RNAi) in tobacco shoots delayed embolism repair compared to the wildtype control and to tobacco plants with reduced expression of *NtPIP1* aquaporin’s. Secchi and Zwieniecki (2010) correlated up-regulation of the *PtPIP1* subfamily in *Populus trichocarpa* (Torr. and Gray) stems with xylem embolism and proposed a continuous embolism/refilling cycle under normal conditions. In *V. vinifera* cv. Grenache, *VvPIP2;1* has been shown to be expressed in petiole tissue and in vessel associated cells (VACs) (Chitarra et al., 2014).

Stomatal closure is thought to minimize water-stress induced cavitation in grapevine (Lovisolo and Schubert, 1998). However, grapevine cultivars are known to vary in their degree of stomatal closure in response to water-stress. Cultivars, such as Grenache, are considered to be near-isohydric, since midday water potential does not decrease substantially under water-stress due to stomatal closure (Schultz, 2003; Vandeleur et al., 2009). In contrast, cultivars such as Shiraz (Syrah) and Chardonnay are more anisohydric with stomata being less sensitive to declining water potential (Schultz, 2003; Soar et al., 2006; Rogiers et al., 2009; Vandeleur et al., 2009). The classification of isohydry and anisohydry for species and cultivars is dependent on many factors including water potential regulation, stomatal behavior, and hydraulic transport under drought conditions (Martínez-Vilalta and Garcia-Forner, 2016). A recent meta-analysis examined factors influencing stomatal conductance in grapevine in response to water availability proposing that there is a continuum of stomatal responses that are dependent upon the scion – rootstock combination and the interaction with different soil types (Lavoie-Lamoureux et al., 2017).

Xylem embolism formation and refilling has been studied extensively in grapevines due to the presence of large xylem vessels that provide a good model for both physiological and molecular studies (Holbrook et al., 2001; Brodersen et al., 2010; Choat et al., 2010). With the sequencing of the grapevine genome (Jaillon et al., 2007; Velasco et al., 2007), and identification of aquaporin families and sub-families (Fouquet et al., 2008; Shelden et al., 2009), grapevine provides an excellent model of a woody perennial fruit tree for studying gene responses to water-stress.

Many studies have investigated the role of aquaporins in root xylem hydraulic function (Lovisolo et al., 2008b; Vandeleur et al., 2009; Perrone et al., 2012); however, there are only a limited number of studies in the shoots (Pou et al., 2013; Chitarra et al., 2014). In this study, we compare the cavitation vulnerability and hydraulic properties of two economically important grapevine cultivars, *Vitis vinifera* L. cv. Grenache (isohydric) and Chardonnay (anisohydric) in response to water-stress (Schultz, 2003; Vandeleur et al., 2009). The objectives of this study were firstly, to compare organ vulnerability to cavitation by measuring xylem cavitation in the petiole and stem, petiole hydraulic conductivity (*K*_petiole_) and xylem anatomical differences between the two cultivars in response to moderate water-stress; secondly, to determine if aquaporin expression was altered in the petioles and leaves both diurnally and in response to water-stress induced cavitation. In order to do this, we measured the transcript abundance of six aquaporin genes, *VvPIP1;1, VvPIP2;1, VvPIP2;2, VvPIP2;3, VvTIP1;1*, and *VvTIP2;1*. *Vitis PIP2* and *TIP* genes have previously been functionally characterized as water conducting channels in *Xenopus* oocytes (Shelden et al., 2009; Vandeleur et al., 2009), however, *VvPIP1;1* only shows water conducting capacity when co-expressed with *VvPIP2* genes (Vandeleur et al., 2009).

## Materials and Methods

### Plant Material

*Vitis vinifera* L. Grenache (clone SA38) and Chardonnay (clone I10V1) 1-year-old rootlings (own roots) (Glen Avon Nursery, Langhorne Creek, SA, Australia) were planted in 4.7 L pots containing a modified University of California (UC) soil mix [61.5% (v/v) sand, 38.5% (v/v) peat moss, 0.50 g L^-1^ calcium hydroxide, 0.90 g L^-1^ calcium carbonate, 100 g per 100 L^-1^ Nitrophoska (12:5:14, N:P:K plus trace elements; IncitecPivot, Melbourne, VIC, Australia) at pH 6.8] and fertilized with 0.08 g L^-1^ (soil) per month of Osmocote Standard (Scotts Australia Pty Ltd., Baulkham Hills, NSW, Australia) as described previously (Shelden, 2008; Shelden et al., 2009). Plants were grown in controlled temperature glasshouses maintained at 25°C day/20°C night with extended light period provided by 1000 watt mercury halide lamps (14 h day/10 h night). Plants were watered to field capacity every 2 days and spur pruned to have two shoots. Pot-grown vines were subjected to a drying cycle to impose water deficit. Plants were watered to field capacity in the evening prior to starting all experiments, then water was withheld for the remainder of the experiment.

### Drought Experiment 1 – Measurement of Acoustic Emissions

Ultrasonic acoustic emissions (UAE) have been used to measure drought-induced cavitation in woody plants including grapevine (Milburn and Johnson, 1966; Tyree and Dixon, 1983; Tyree et al., 1984; Ikeda and Ohtsu, 1992; Kikuta et al., 1997; Kikuta et al., 2003; Johnson et al., 2009). A cavitation event occurs in the xylem vessels due to increased tension as a result of drought, resulting in a rapid relaxation of energy that produces an acoustic emission of energy (AE). Ultrasonic acoustic emissions were measured using an acoustic monitoring system (Model 4615 DSM, Physical Acoustic Corporation, Princeton Junction, NJ, United States) with I151 sensors. Signals were amplified in the range of 150–400 kHz. At least five individual plants grown in the glasshouse were monitored over the growing season for each variety. Sensors were positioned on the basal portion of the plant between nodes two and six on fully mature leaves. One sensor was clamped to the middle of an internode and one to a petiole. A thin layer of silicon grease was put onto the transducer to allow better acoustic contact with the plant. The plant was watered to field capacity at the start of the experiment and UAE’s recorded continuously over the drought period until wilting point was reached. As Chardonnay reached wilting point before Grenache, measurements were taken over a longer period for Grenache. The cumulative UAEs (cUAE) were determined and plotted against time of water-stress. In addition, cUAEs were normalized relative to the maximum number of cavitated vessels observed at extreme dehydration in each organ (petiole and stem) and plotted against the mean leaf water potential (ψ_L_). A sigmoidal dose-response curve (variable) slope was fitted to the data using GraphPad Prism^®^ Version 4.0.

$$Y=\text{Bottom}+(\text{Top}-\text{Bottom})/(1+10^{\text{LogEC}50-X})$$

The cavitation threshold value (ψ_10_) is the leaf water potential determined at the point at which cavitation is triggered and is taken as 10% of the maximum UAEs (Salleo et al., 1996; Nardini et al., 2001). Both ψ_10_ and ψ_50_ were determined for the stem and petiole of at least three plants of each cultivar. Significance between means were determined with an unpaired *t*-test using GraphPad Prism^®^.

### Leaf Water Potential

Leaf water potential (ψ_L_) measurements were made using the PSYPRO^TM^ data logger with L-51(A)-SF leaf psychrometer sensors (Wescor, Inc., Logan, UT, United States). Psychrometers were calibrated with NaCl solutions as described by Campbell and McInnes (1999). Leaf psychrometers were positioned on four basal leaves surrounding the acoustic sensors. Prior to attachment, the leaf cuticle was removed from the abaxial side of the leaf with 1200 grit sandpaper as described by Campbell and McInnes (1999). The scan sequence program was set as follows: cooling current time 15 s, measurement period seconds 20 s, delay seconds after cooling 5.2 s, and read average 6 s. For each psychrometer a reading was taken every 15 min over the duration of the experiment. Where indicated, leaf water potentials were also measured with a pressure chamber (PMS Instruments, Albany, OR, United States). A calibration performed of leaf psychrometers versus pressure chamber yielded a linear relationship (Supplementary Figure S1). The use of psychrometers was preferential as it was non-destructive and allowed continuous *in situ* monitoring of ψ_L_ over the experiment duration.

### Xylem Anatomy

Transverse sections of fresh petioles were stained with toluidine blue O and examined to determine the vessel diameter of mature xylem vessels in Chardonnay and Grenache vines. Hand sections were made using a single razor blade in the middle of the petiole. Sections were stained with 0.05% v/v toluidine blue O for 1 min, rinsed with distilled water and mounted onto slides. Sections were viewed under a light microscope (Zeiss Axiophot Pol Photomicroscope, Oberkochen, Germany). Images were captured using a Nikon DXM1200F digital Video (Coherent Life Sciences) and Nikon ACT-1 software. The mean xylem vessel diameter was determined for each variety by sampling petioles from four vines and measuring the diameter of xylem vessels per petiole segment. The weighted hydraulic diameter (*d*_hyd_) was calculated as:

$$d_{\text{hyd}}=\sum r^{5}/\sum r^{4}$$

where *r* is the radius of the vessel, as described by Sperry et al. (1994).

### Drought Experiment 2

Plants were grown in controlled temperature glasshouses as described above and watered to field capacity the evening prior to beginning observations. Four individual plants were harvested at the following time points: 6:00, 11:00, 16:00, and 21:00 h on each sampling day (well-watered and water-stressed) (16 plants in total for each cultivar). The same plants were sampled for each time point under well-watered and water-stressed conditions. The 6:00 h time point was harvested in the dark and is referred to in the text as predawn ψ_L_, and 11:00 h as midday ψ_L_.

One leaf from each plant was taken to measure ψ_L_ at each time point with the Scholander pressure chamber (PMS Instruments, Albany, OR, United States). Stem water potential (ψ_s_) was determined by covering a leaf on each plant with a plastic bag wrapped in aluminum foil for 1 h prior to measurement with the Scholander pressure chamber (Begg and Turner, 1970). One petiole with leaf attached was harvested for measurement of hydraulic conductance and Percent Loss of Conductance (PLC). One leaf per plant was used to measure transpiration and stomatal conductance (*g*_S_) with a LI-6400 Portable Photosynthesis System (LI-COR, Lincoln, NE, United States) at 11:00and 16:00 h on the days of sampling. Over the course of the experiment, midday stomatal conductance (*g*_S_) was measured on all plants using a porometer (Delta T AP4; Delta-T Devices Ltd., Cambridge, United Kingdom).

On the first sampling day (well-watered), two petioles and corresponding leaves were sampled from nodal positions three to five at the basal portion of the stem at all time points for aquaporin expression analysis. Petioles were cut parallel to the shoot axis with a sharp razor blade and the petiole detached from the base of the leaf. Tissues were wrapped separately in aluminum foil and immediately snap frozen in liquid nitrogen. Water was withheld from plants for the next 3 days at which time the same plants were sampled again (water-stressed, when midday leaf water potentials were predicted to be approximately -1.5 MPa). The same plants were sampled for each time point under well-watered (WW) and water-stress conditions (WS).

### Petiole Hydraulic Conductivity and Percent Loss Conductance (PLC)

The petiole specific hydraulic conductivity (*K*_h_, m^4^ s^-1^ MPa^-1^) and PLC was measured using the XYL’EM Embolism Meter apparatus (Bronkhorst, France). The XYL’EM apparatus was equipped with a pressure transducer and two flow meters (Liquiflow, Instrutec; 5 and 50 g h^-1^). Degassed glass distilled water (Labglass Cascade, Graintech, Australia) was used to fill the captive air tank. Petioles were cut parallel to the node submerged under MilliQ water and immediately attached to luer tubes that were connected to the XYL’EM apparatus. The leaf blade was removed from the petiole after connection to the luer tube. To obtain *K*_init_, petioles were perfused with degassed distilled water at 4 kPa. Petioles were then flushed at 0.15 MPa for 1 min and *K*_final_ determined. The water flow (F; m^3^ s^-1^) entering the petiole was measured when exposed to a positive pressure (P; MPa) of 0.15 MPa and recorded when flow rate was stable (usually between 1 min). PLC was computed:

$$\text{PLC}=100^{*}(1-K_{\text{init}}/K_{\text{final}})$$

On occasions, *K*_final_ values were lower than *K*_initial_ computing a negative PLC value. This can result from blockage of the xylem vessels during measurement (Cochard et al., 2013). Where this occurred, these values were recorded as zero PLC in the analysis.

The petiole specific hydraulic conductivity, *K*_Pet_h_, was computed as:

$$K_{\text{Pet}\_\text{h}}=(F\times L)/P$$

Where *L* is the length of the petiole segment (m).

Leaf area (LA; m^2^) was measured with a leaf area meter (AM200, ADC Bioscientific Ltd., Herts, England). Leaf specific conductivity (*K*_Pet_LA_) was determined by:

$$K_{\text{Pet}\_\text{LA}}=K_{\text{Pet}\_\text{h}}/\text{LA}$$

The mean *K*_Pet_h_ was calculated from averaging *K*_Pet_h_ values from all time points of well-watered vines.

### Total and Poly(A)^+^ RNA Isolation cDNA Synthesis

Total RNA was isolated from petiole tissue as described previously (Shelden et al., 2009) and treated with RNase free DNase 1 (Ambion, Melbourne, VIC, Australia). First strand cDNA synthesis from normalized total RNA was synthesized using the iScript^TM^ cDNA synthesis kit (Bio-rad, Hercules, CA, United States) from 1 μg of total RNA. Leaf total RNA was extracted using the Spectrum Plant Total RNA Kit (Sigma) and cDNA synthesized using the Superscript III First Strand Kit (Invitrogen). Quantity and purity of total RNA was determined with a Nanodrop Spectrophotometer ND-1000 (Biolab Ltd., Australia).

### Quantitative Real-Time Polymerase Chain Reaction (QRT-PCR)

Gene specific primers (Sigma–Aldrich, Castle Hill, NSW, Australia) were designed with Primer3^1^ (accessed July 2006) to previously described aquaporin cDNAs, *VvPIP1;1, VvPIP2;1, VvPIP2;2, VvPIP2;3, VvTIP1;1*, and *VvTIP2;1* (Shelden et al., 2009), in regions with highest sequence divergence (Supplementary Table S1). Primers were designed to amplify amplicons between 110 and 230 bp in length with *T*_m_ between 58 and 65°C, and GC content not higher than 55%.

A 2 × mix of KAPA SYBR^®^FAST qPCR (KAPA Biosystems) was used for all real-time PCR reactions. The reaction mix contained 2 × KAPA Master Mix, 10 μM of each primer and an amount of cDNA template equivalent to 15 ng of total RNA. Twenty microliter reactions were used and each reaction was performed in duplicate. Thermocycling was conducted in a QuantStudio 12K Flex Real-Time PCR system (Life Technologies): 95°C for 3 min, 40 cycles consisting of 95°C for 1 s, 55°C for 20 s, and 72°C for 10 s. Prior to melt curve analysis a final denaturation step at 95°C for 30 s was performed. Melt curve analysis was performed between 57 and 97°C at 0.5°C increments for 30 s. To ensure single-product amplification, melt curve analysis was performed by heating the PCR products for 40 cycles starting at 52°C and increasing by 0.5°C per cycle with continuous fluorescence detection.

Amplification efficiencies varied between 95 and 100%. To confirm correct amplicon, PCR products were sequenced using Dye Terminator 3 (Applied Biosystems, Foster, CA, United States) and analyzed by the Institute of Medical and Veterinary Sciences (Adelaide, SA, Australia). Data were normalized with *VveELFγ, VvACT, and VvUbq* that have been shown to be constitutively expressed in grapevine (Deluc et al., 2006, 2008). Standard curves were generated for each gene using gene specific primers and 10-fold serial dilutions of purified PCR amplified gene specific products. The correlation coefficient and PCR amplification efficiency (E) was determined for each primer set. Relative changes in gene expression were determined using the Pfaffl method (Pfaffl, 2001). The values were calculated relative to each reference gene and then the geometric mean was determined (Vandesompele et al., 2002). Outliers were removed using the ROUT algorithm (Motulsky and Brown, 2006). The Log2 ratio was calculated for diurnal expression as 11:00/6:00 h, 16:00/6:00 h, and 21:00/6:00 h and for water-stress treatment as WS/WW. Data are presented as the mean normalized expression of four biological representatives (petioles and leaves taken from individual plants) ± SE, each with two technical replicates.

### Statistical Analysis

Statistical analysis was performed with GraphPad Prism^®^(GraphPad Software Inc, San Diego, CA, United States). One-way ANOVA and two-way ANOVA were performed to test differences between experimental groups. Two tailed unpaired *t*-tests were used to compare mean values. The correlation matrix was performed using RStudio software (Version 1.0.153^2^). Hydraulic conductivity and QPCR data were log normal transformed prior to performing correlation in RStudio.

## Results

### Drought Experiment 1: Leaf Water Status and Cavitation Vulnerability

Leaf water potential measurements were made continuously with leaf psychrometers over the course of the drought experiments on both Chardonnay and Grenache vines. A linear regression of ψ_L_ measured with the pressure chamber and psychrometers showed a good correlation with no significant difference observed (Supplementary Figure S1). The pressure chamber tended to measure lower values for ψ_L_ particularly at higher water potentials than psychrometers as has been reported in other species (Martinez et al., 2011). Psychrometers recorded water potential every 15 min, thus oscillations were observed throughout the day (Supplementary Figure S2) indicating the continuously changing water status of the leaf most likely due to localized changes in water availability and stomatal conductance (During and Loveys, 1996). The mean predawn ψ_L_ of well-watered Chardonnay vines was -0.26 MPa and in Grenache -0.29 MPa (**Table 1**). In water-stressed Grenache vines the predawn and midday ψ_L_ were significantly higher (-0.7 and -1.05 MPa, respectively) than Chardonnay vines (-1.17 and -1.44 MPa) in response to water-stress.

**Table 1:** Drought experiment 1 predawn (ψ_L_Predawn_, MPa) and midday leaf water potential (ψ_L_Midday_, MPa) for Chardonnay and Grenache vines used for cavitation analysis under well-watered (WW) and water-stressed (WS) conditions.

Cultivar	WW ψ_L_Predawn_	WW ψ_L_Midday_	WS ψ_L_Predawn_	WS ψ_L_Midday_
Chardonnay	-0.26 ± 0.06^a^	-0.31 ± 0.01^b^	-1.17 ± 0.01^c^	-1.44 ± 0.01^d^
Grenache	-0.29 ± 0.10^a^	-0.34 ± 0.11^b^	-0.70 ± 0.09^e^	-1.05 ± 0.06^f^

*Plants were grown in control temperature glasshouses, watered to field capacity the evening prior to WW measurements being recorded and water withheld until wilting point. WS is reported after 96 h of water withheld. Leaf water potential was recorded continuously with psychrometers on the same plant as ultrasonic acoustic emissions (UAEs) were recorded. Mean values of three independent experiments ± SEM (*n* = 3) are shown. Superscript letters show significance between both cultivars and time of day (two-way ANOVA, Bonferroni post-test, *p* > 0.05).*

**FIGURE 1:**
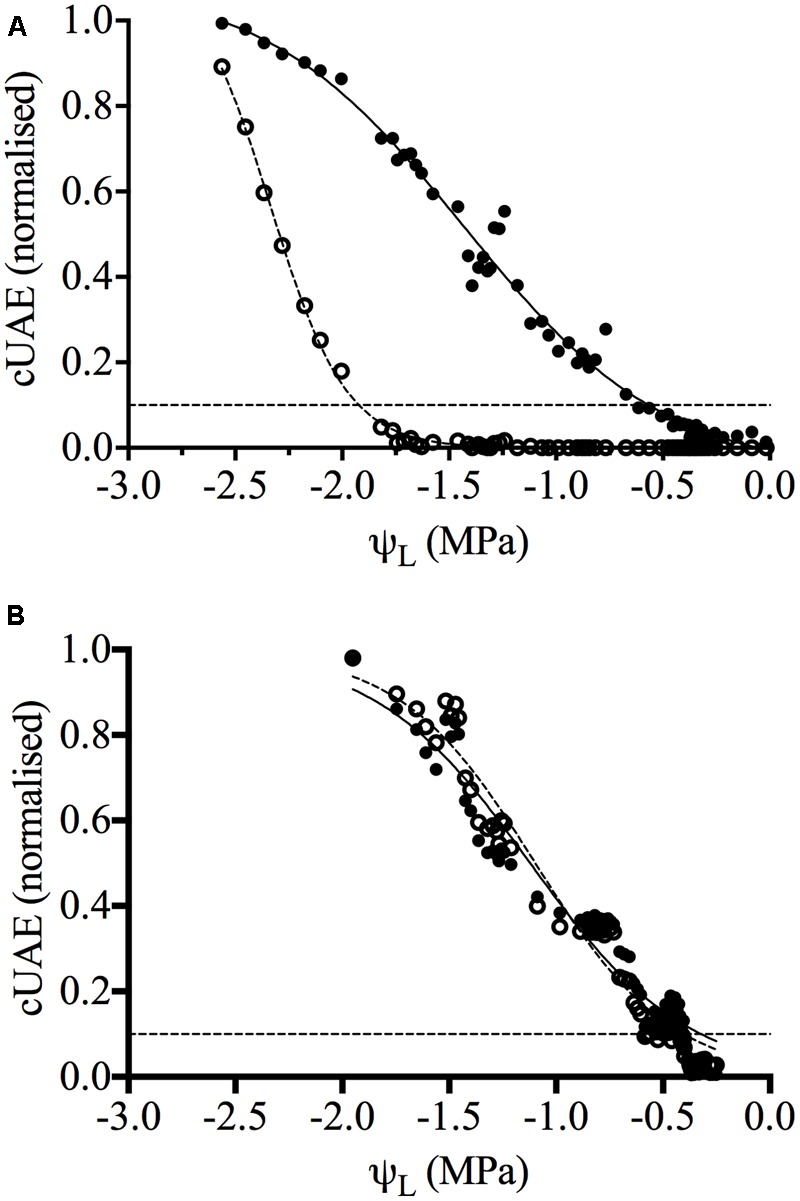
Vulnerability curves for Chardonnay **(A)** and Grenache **(B)** grapevines. Data shown is the normalized cumulative ultrasonic acoustic emissions (cUAE) plotted against mean leaf water potential for both the petiole (filled circles, bold line) and stem (open circles, dotted line) (*n* = 1). Curves were generated by fitting a sigmoidal dose-response curve (variable slope) to the data using Graphpad Prism software. The hillslope (*r*^2^ in brackets) for Chardonnay petiole and stem is –0.9 (0.99) and –2.4 (0.99), respectively, and for Grenache petiole and stem –1.2 (0.94) and –1.4 (0.97), respectively. The dotted black line represents 10% of the maximum cUAEs.

Cavitation was measured in the internodes and the middle of petioles of both Chardonnay and Grenache vines, by the detection of UAEs using specialized microphones (Supplementary Figure S2). Vulnerability curves were generated by plotting the cUAEs against the leaf water potential, for three independent drying experiments for both Chardonnay and Grenache (**Figure 1**). The number of emissions per day increased over time as the water-stress became more severe. In well-watered Chardonnay vines, cavitation was only detected in the petiole and not in the stem internode (**Figure 1A**). As the water-stress increased (ψ_L_ values ∼-1.5 MPa), UAEs were detected in both the stem and petiole (**Figure 1A**). In Grenache, UAEs were detected simultaneously in both the petioles and stems of well-watered vines (**Figure 1B**). From the vulnerability curves, it is possible to determine the ψ_CAV_ threshold value, taken as 10% of the maximum cUAEs, for the point at which cavitation is triggered (Salleo et al., 1996; Nardini et al., 2001). In Grenache vines, the petiole and stem had very similar ψ_10_ values of -0.13 and -0.19 MPa, respectively (**Table 2**). In Chardonnay vines, the leaf water potential (ψ_10_) at which cavitation was triggered in the petioles and stems was significantly different (*p* < 0.05), with threshold ψ_10_= -0.53 and -1.81 MPa for the petioles and stems, respectively. There was no significant difference between cultivars in threshold ψ_10_ for petioles, however, the stem ψ_10_ was significantly different. Leaf water potential that induced 50% loss of conductance was significantly different between petioles and stems in Chardonnay (ψ_50Petiole_ = -1.14 and ψ_50Stem_= -2.24 MPa) but not in Grenache (ψ_50Petiole_ = -0.73 and ψ_50Stem_= -0.78 MPa). There was no significant difference between cultivars in ψ_50Petiole_, however, ψ_50Stem_ was significantly more negative in Chardonnay than Grenache.

**Table 2:** Leaf water potential for threshold cavitation (ψ_10_) and 50% loss of conductance (ψ_50_) assayed by acoustic emissions (AEs) in the petioles and stems of Chardonnay and Grenache potted vines.

Cultivar	ψ_10Petiole_ (MPa)	ψ_10Stem_ (MPa)	ψ_50Petiole_ (MPa)	ψ_50Stem_ (MPa)
Chardonnay	-0.53 ± 0.04^a^	-1.81 ± 0.23^b^	-1.13 ± 0.13^a^	-2.24 ± 0.23^b^
Grenache	-0.13 ± 0.10^a,c^	-0.19 ± 0.^11c^	-0.73 ± 0.2^a^	-0.78 ± 0.18^a^

*Ψ_*10*_ is determined as the leaf water potential for which 10% of the maximum AE. Ψ_*50*_ is the leaf water potential that induces 50% loss of hydraulic conductance. Data is the mean ± SEM of at least three independent experiments. Superscript letters indicate significance between cultivar and organ (*t*-test, *p* < 0.05).*

### Drought Experiment 2: Leaf Water Status, Hydraulic Conductivity, and Xylem Anatomy

**FIGURE 2:**
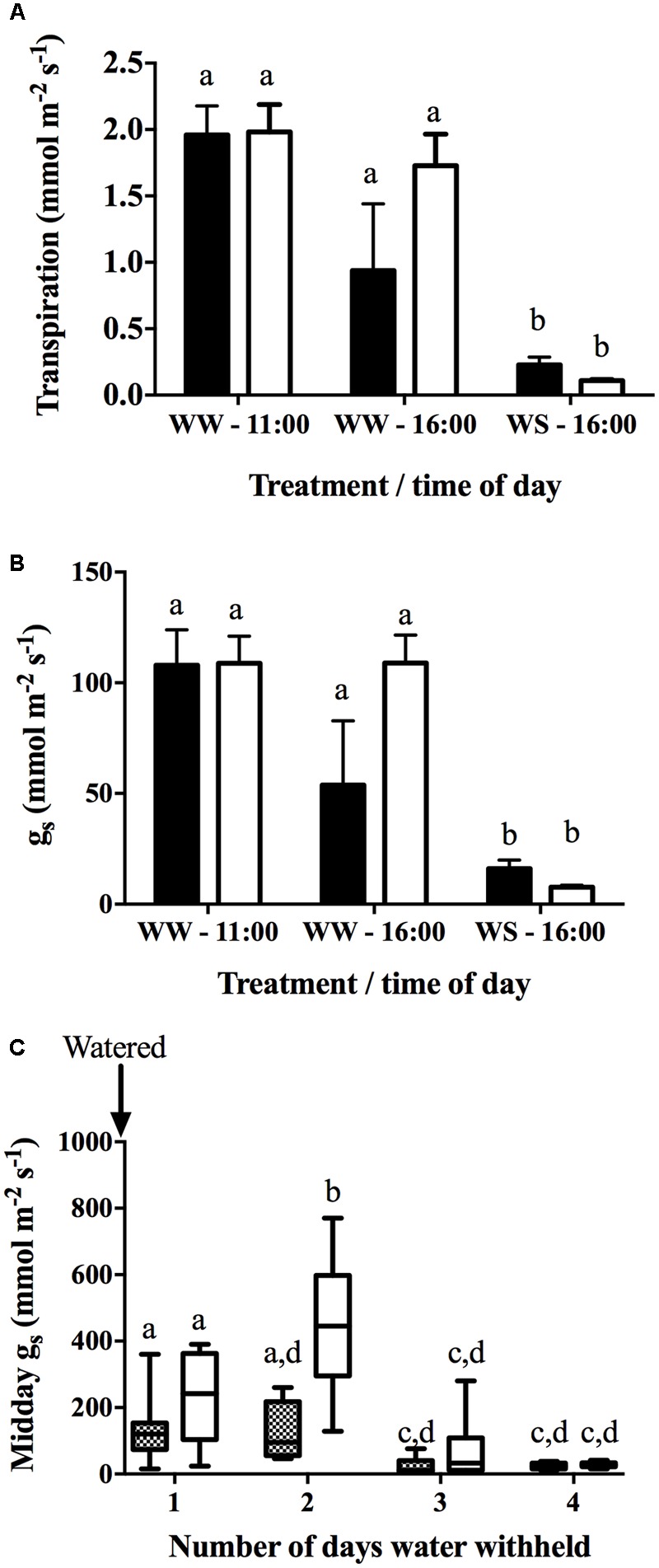
Stomatal conductance (*g*_S_) **(A)** and transpiration (*E*) **(B)** were measured at 11:00 and 16:00 h on well-watered (WW) (D1) plants and at 16:00 h of water-stressed (WS) (D4) plants with the Licor (*n* = 4). Plants were watered to field capacity the evening prior to the first day of sampling (D1 – WW) and water was withheld for 4 days (D4 – WS). Data shows the mean ± SEM. Midday leaf stomatal conductance (*g*_S_) **(C)** measured on Grenache and Chardonnay plants using the porometer over the duration of water withholding experiment. Data shows the mean ± min/max value (*n* = 9–16). Significant differences between cultivars and time points are indicated by different letters (*p* < 0.05).

In Drought Experiment 2, leaf and stem water potential displayed a diurnal pattern for both varieties (**Table 3**). In Chardonnay, a significant difference was observed between well-watered and water-stress at each time point except for predawn (6:00 h) after 72 h of withholding water. In Grenache, there was no significant difference between well-watered and water-stressed plants except at 21:00 h. No significant differences in ψ_L_ were observed between cultivars under well-watered and water-stressed conditions (**Table 3**). The stem water potential was significantly different between well-watered and water-stressed conditions for both Chardonnay and Grenache at each time point (**Table 3**). A significant difference in ψ_S_ was observed at 16:00 h between water-stressed Chardonnay (-1.38 MPa) and Grenache (-1.18 MPa) with Grenache maintaining a less negative ψ_s._ Both *E* and *g*_S_ were significantly decreased in water-stressed Chardonnay and Grenache compared to well-watered vines at 16:00 h (**Figures 2A,B**). Midday stomatal conductance (*g*_S_) measurements showed Chardonnay had a significantly higher *g*_S_ than Grenache on D2 after water was withheld (**Figure 2C**). In both Chardonnay and Grenache, midday *g*_S_ decreased significantly as the vines became more water-stressed after 3 days of water withheld. Both stem and leaf water potential were strongly positively correlated with *E* and *g*_S_ in Chardonnay, however, only stem water potential correlated in Grenache.

**Table 3:** Drought Experiment 2 leaf water potential (ψ_L_, MPa) and stem water potential (ψ_S_, MPa) for Chardonnay and Grenache WW and WS vines.

	Chardonnay		Grenache	
	ψ_L_ (MPa)		ψ_L_ (MPa)	
Time	WW	WS	WW	WS
6:00	-0.2 ± 0.03^a^	-0.5 ± 0.05^a^	-0.3 ± 0.02^a^	-0.5 ± 0.06^a^
11:00	-0.7 ± 0.03^c^	-1.0 ± 0.16^d^	-0.9 ± 0.04^b,c^	-1.0 ± 0.06^b,e^
16:00	-1.0 ± 0.08^e^	-1.5 ± 0.04^f^	-1.1 ± 0.18^b^	-1.3 ± 0.07^b,e^
21:00	-0.3 ± 0.02^a^	-1.0 ± 0.02 ^d^	-0.6 ± 0.04^a,c^	-1.2 ± 0.04^e^

	**Ψ_S_ (MPa)**		**Ψ_S_ (MPa)**	
**Time**	**WW**	**WS**	**WW**	**WS**

6:00	–	–	–	–
11:00	-0.5 ± 0.03^a^	-0.8 ± 0.03^b^	-0.6 ± 0.02^a^	-0.9 ± 0.06^b^
16:00	-0.6 ± 0.07^a^	-1.4 ± 0.05^c^	-0.8 ± 0.07^b^	-1.2 ± 0.06^c^
21:00	-0.4 ± 0.03^a^	-1.2 ± 0.07^c^	-0.5 ± 0.0^a^	-1.1 ± 0.02^c^

*Plants were grown in control temperature glasshouse and measurements conducted at the time of tissue sampling for QPCR analysis. Plants were watered to field capacity the evening prior to WW measurements, and water withheld for 72 h for WS measurements. Leaf water potential and stem water potential measurements were taken with the pressure chamber. Mean ± SEM (*n* = 4). Superscript letters indicate significance differences (two-way ANOVA, Bonferroni post-test, *p* < 0.05).*

**FIGURE 3:**
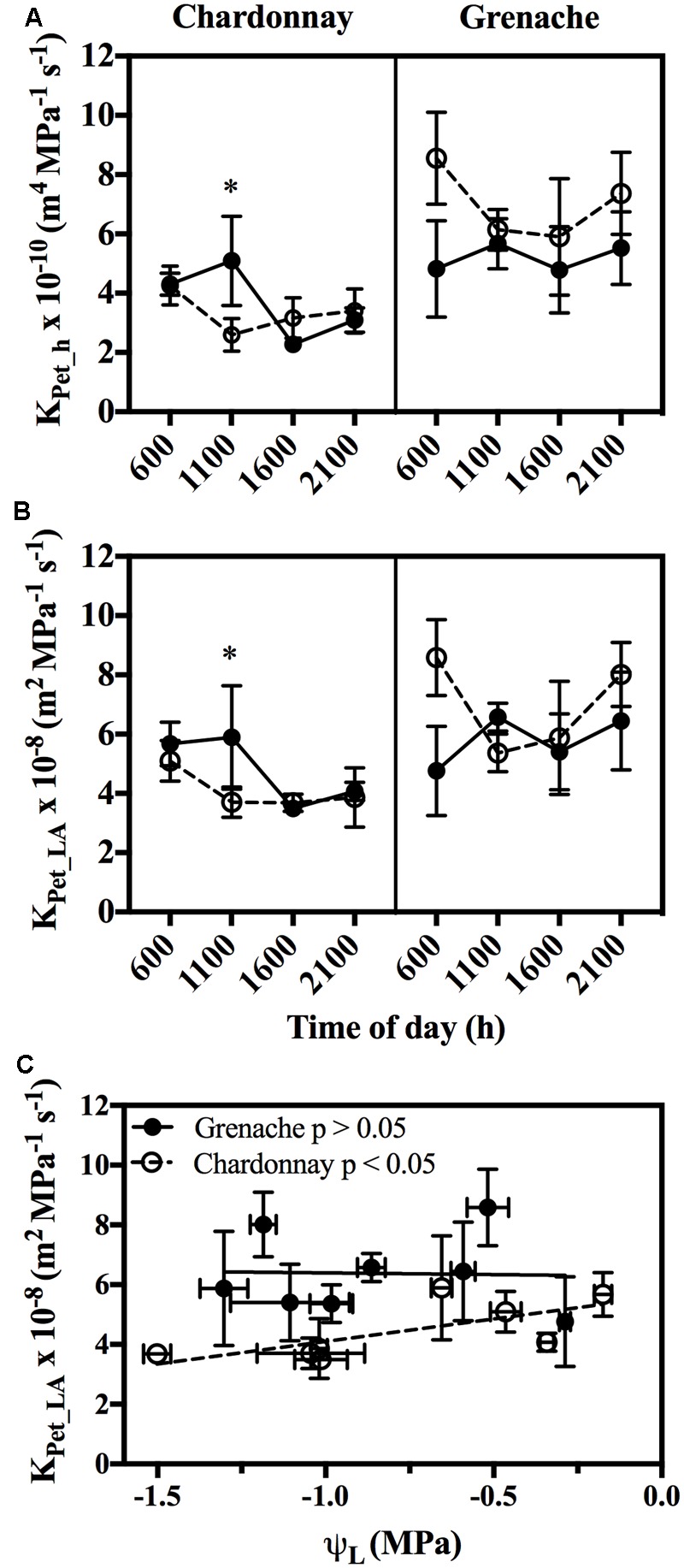
Mean petiole specific hydraulic conductivity (*K*_Pet_h_) **(A)** and mean leaf area specific conductivity (*K*_Pet-LA_) **(B)** over a diurnal period in WW (filled circles, bold line) and WS (open circles, dotted line) Chardonnay and Grenache vines. The relationship between *K*_Pet-LA_ and leaf water potential (ψ_L_) for Chardonnay and Grenache was determined by fitting a linear regression (significant *p* < 0.05) **(C)**. Data shows the mean ± SEM (*n* = 4). Significant differences between WW and WS are indicated by ^∗^ (*p* < 0.05).

**FIGURE 4:**
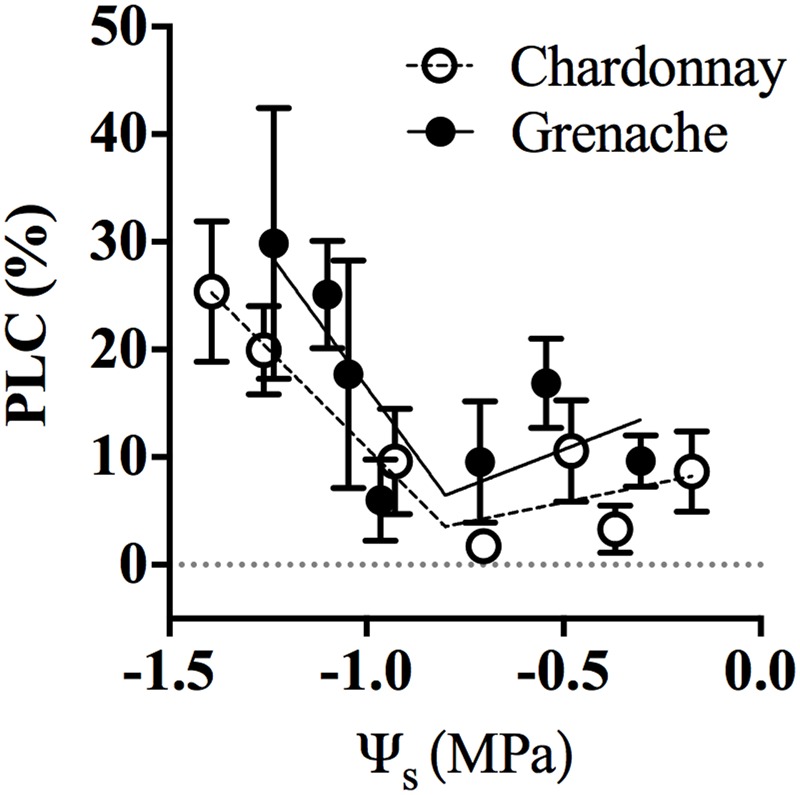
Percent loss of conductance (PLC) in response to stem water potential (ψ_S_) for Chardonnay (open circles) and Grenache petioles (filled circles). Well-watered and water-stressed data were combined for each cultivar and ψ_S_ ranked. Data was segmented in to approximately equal steps in ψ_S_ and means ± SEM calculated for each category (*n* = 3–9 for each point). There were 32 separate observations for Chardonnay and 31 for Grenache. For each cultivar data was fitted with a segmental linear regression; the break point for both = –0.9 MPa. The first slope is not significantly different from zero and the intercept is also not significantly different from zero (i.e., zero PLC above –0.8 MPa). Below –0.8 MPa the PLC increases with a slope of 14.1 and 7.5% PLC per MPa for Grenache and Chardonnay, respectively, however, these are not significantly different.

Hydraulic conductivity measurements were conducted on excised petioles from Chardonnay and Grenache vines grown in a temperature-controlled glasshouse. In response to well-watered and water-stressed conditions, no significant diurnal changes in petiole hydraulic conductivity (*K*_Pet_h_ and *K*_Pet_LA_) were evident in either cultivar (Two-way ANOVA, *p* > 0.05) (**Figures 3A,B**). Water-stress significantly decreased petiole *K*_Pet_h_ and *K*_Pet_LA_ for Chardonnay at 11:00 h. There were no significant differences in *K*_Pet_h_ or *K*_Pet_LA_ for well-watered and water-stressed Grenache vines. K_Pet_LA_ showed a significantly linear decline with decreasing ψ_L_ in Chardonnay but not in Grenache (**Figure 3C**). Both *K*_Pet_h_ and *K*_Pet_LA_ were higher in Grenache petioles than Chardonnay, although this difference was not significant. The mean *K*_Pet_h_ was 3.7 × 10^-10^ m^4^ MPa^-1^ s^-1^ and 5.2 × 10^-10^ m^4^ MPa^-1^ s^-1^ in Chardonnay and Grenache, respectively. PLC significantly increased under water-stress in Grenache petioles with maximum PLC 35% at 16:00 h when both ψ_L_ and ψ_S_ were most negative (Supplementary Figure S3). In Chardonnay, maximum PLC was 23%, however, there was no significant difference at any time point in response to water-stress. When PLC was plotted against stem water potential no difference in slope was observed between cultivars (**Figure 4**). When ψ_S_ > -0.80 MPa, the fitted slope was not significantly different from zero, however, ψ_S_ < -0.80 MPa, PLC increased with a slope of 14.1 and 7.5% PLC per MPa for Grenache and Chardonnay, respectively, however, these were not significantly different.

**FIGURE 5:**
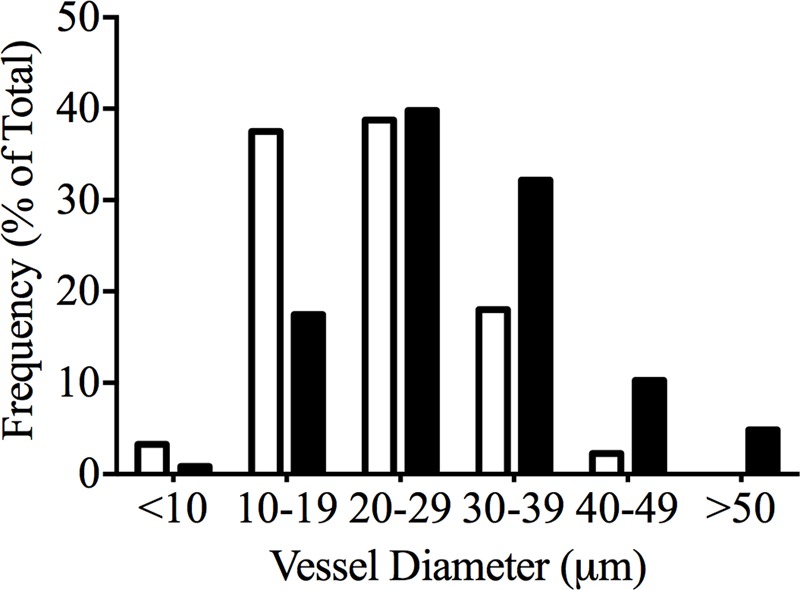
Relative frequency of different xylem vessel diameters in mature Chardonnay (black bars) and Grenache (white bars) petioles calculated from transverse sections of four individual petioles.

Xylem vessel diameters were measured on mature petioles of Chardonnay and Grenache. The frequency of petioles with xylem diameter between 20 and 29 μm was similar for both Chardonnay and Grenache; however, xylem vessel diameters greater than 30 μm were frequently measured in Grenache petioles (**Figure 5**). In Grenache petioles, the average xylem vessel diameter was significantly larger than Chardonnay petioles, as were both the minimum and maximum xylem diameters (**Table 4**). Chardonnay had a weighted hydraulic diameter (*d*_hyd_) of 32.1 μm and Grenache had a *d*_hyd_ of 44.1 μm calculated according to Sperry et al. (1994). Thus, based on *d*_hyd_, Chardonnay petioles would have less capacity to conduct water compared to Grenache.

**Table 4:** Minimum, maximum and mean xylem vessel diameter (μm) for mature Chardonnay and Grenache petioles for plants grown in a growth chamber.

	Min. vessel diameter (μm)	Mean vessel diameter (μm)	Max. vessel diameter (μm)
Chardonnay	5.0	20.7 ± 0.7^a^	55
Grenache	7.5	30.5 ± 3.5^b^	65

*Vessel diameters (μm) were obtained from transverse sections of petioles harvested from four individual plants. Data for vessel diameter is the mean ± SEM (*n* = 4 petioles). Superscript letters indicate significance between cultivars (*p* < 0.05).*

### Diurnal Regulation of AQP Expression under Well-Watered Conditions

To investigate if aquaporin gene expression in the petioles and leaves correlated with physiological parameters we monitored the expression of six genes *VvPIP1;1, VvPIP2;1, VvPIP2;2, VvPIP2;3, VvTIP1;1*, and *VvTIP2;1* using QPCR over a diurnal cycle of well-watered and water-stressed Chardonnay and Grenache vines. Transcripts of all these genes have previously been detected in other vegetative tissues in grapevine (stem, tendrils, leaves, and roots) and thus are not specific to the petiole and leaves (Shelden, 2008; Vandeleur et al., 2009). Petioles and leaves were sampled from four independent plants used for physiological measurements, but due to the extremely low expression of some aquaporin genes (undetectable by QPCR), some data points represent less biological representatives. A major problem linked to studying gene expression in conductive tissues (i.e., petioles) is the low transcript abundance of mRNA (Sakr et al., 2003).

**FIGURE 6:**
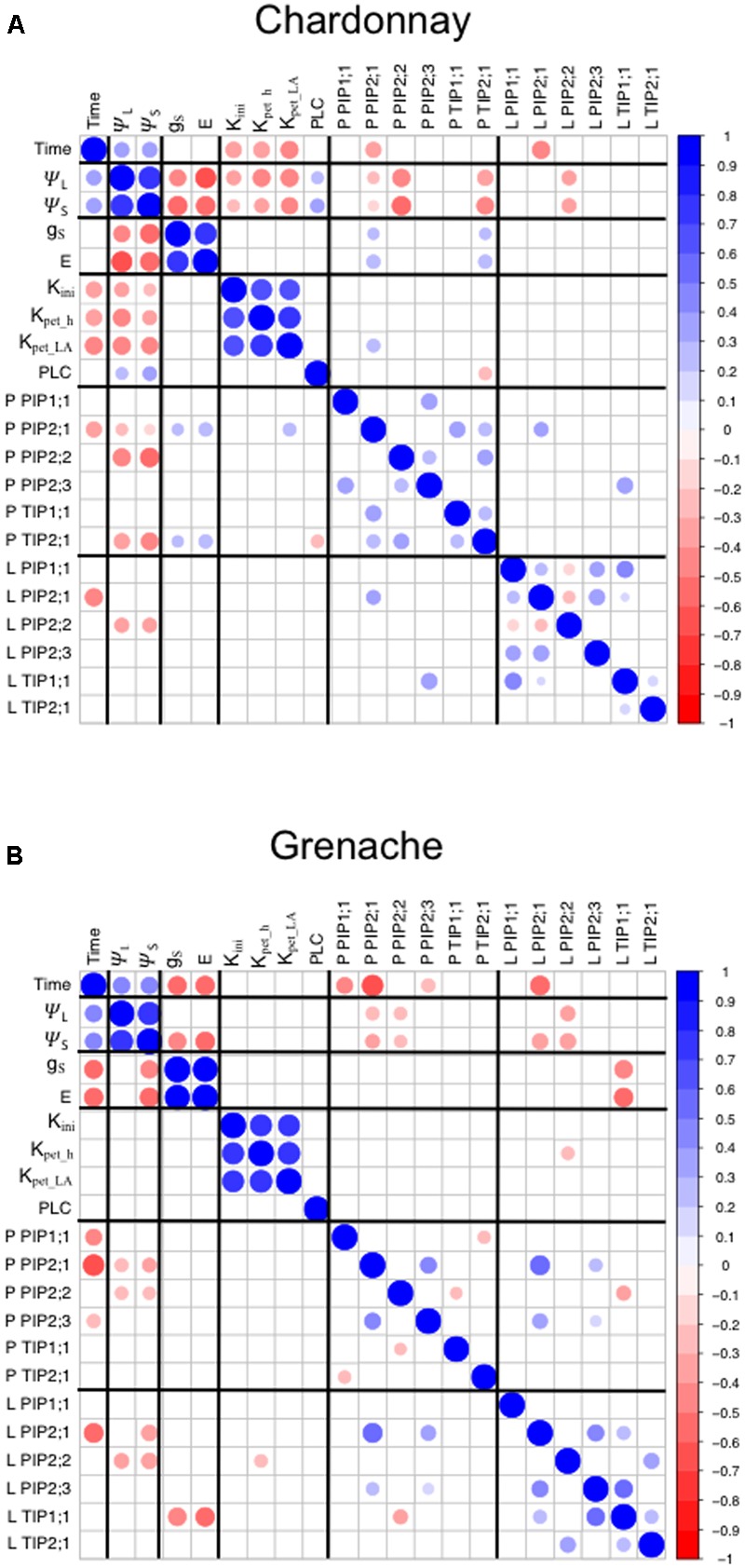
Correlation matrix of physiological parameters and aquaporin gene expression in Chardonnay **(A)** Grenache **(B)**. All data was obtained from Drought Experiment 2. Leaf and stem water potentials were assumed to be the same for 6:00 h (predawn). Only significant Pearson correlation coefficients are shown. P-petiole and L-leaf for aquaporin gene expression.

To analyze the diurnal/circadian response of aquaporin gene expression under well-watered conditions, the expression data at each time point was compared to the expression levels predawn (6:00 h) (**Table 5**). Several aquaporin genes examined showed evidence of diurnal regulation of gene expression. The expression of *VvPIP2;1* negatively correlated with time in the petioles and leaves of both cultivars (**Figures 6A,B**). *VvPIP1;1*_petiole_ and *VvPIP2;3*_petiole_ negatively correlated with time in Grenache only (**Figure 6B**). The diurnal expression pattern of *VvPIP2;1* was similar for both cultivars and tissues examined (petioles and leaves) with expression highest predawn, subsequently decreasing over the day with expression levels lowest at 21:00 h (Supplementary Figures S4–S7). Expression of *VvPIP2;1* was significantly decreased at all time points in Chardonnay petioles and at 16:00 h and 21:00 h in all other samples compared with predawn levels (6:00 h). The mean normalized expression of *VvPIP2;1* was significantly higher in Grenache petioles than Chardonnay petioles (Supplementary Figures S4, S5).

**Table 5:** Diurnal expression of aquaporin genes in Chardonnay and Grenache petioles and leaves.

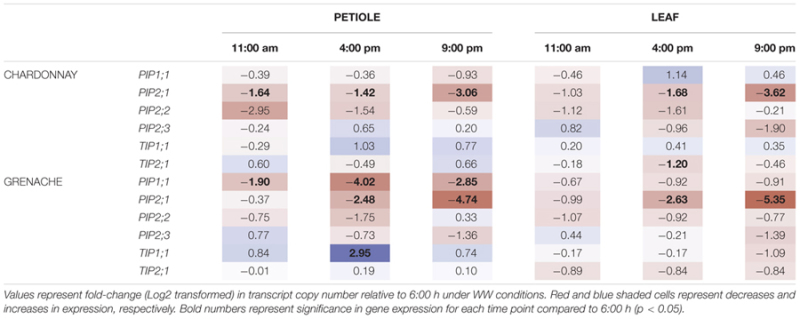

Expression of *VvPIP2;2* in Chardonnay and Grenache was highest predawn and decreased throughout the day, however, the response was not significant. In contrast to *VvPIP2;1*, expression levels of *VvPIP2;2* increased at 21:00 h to values similar to predawn levels. There were no significant changes in expression of *VvPIP2;3* in the petioles and leaves for either cultivar compared with 6:00 h (**Table 5**).

No significant diurnal response was observed in the petioles or leaves of either cultivar for *VvTIP2;1* (Supplementary Figures S4–S7) although a significant decrease in expression was observed at 16:00 h in Chardonnay leaves. Expression of *VvTIP1;1* significantly increased in Grenache petioles at 16:00 h compared to predawn levels but no other changes were observed. No diurnal response for *VvTIP1;1* was observed in Chardonnay petioles or leaves of either cultivar.

### Transcriptional Regulation of Aquaporins in Response to Water-Stress

To determine the transcriptional response of aquaporin’s to a moderate water-stress, petioles and leaves were harvested from Chardonnay and Grenache when midday ψ_L_ (11:00 h) was approximately -1.0 MPa. Chardonnay and Grenache differed in their response to water-stress, with expression of aquaporin’s in Chardonnay petioles tending to be more down-regulated than in Grenache petioles compared to well-watered expression (**Table 6**).

**Table 6:** Aquaporin gene expression in response to water-stress treatment.

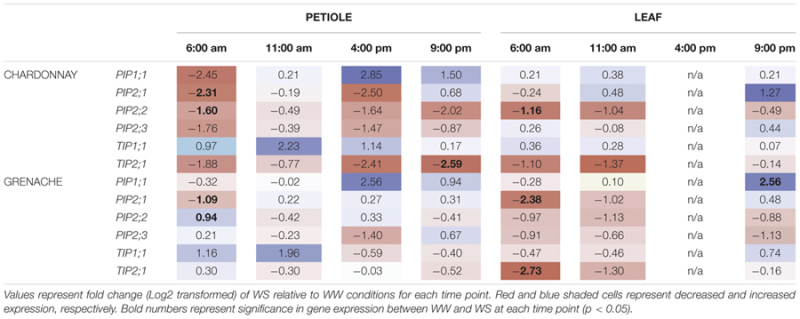

In response to water-stress, there was a significant down-regulation of *VvPIP2;1* at 6:00 h (predawn) in both Chardonnay and Grenache petioles and in the leaves of Grenache (**Table 6**). Mean normalized expression was highest predawn for both cultivars and tissues under water-stress (Supplementary Figures S4–S7). Both *VvPIP2;1* and *VvPIP2;2* were strongly down-regulated in Chardonnay petioles but not Grenache petioles in response to water-stress. In Chardonnay, *VvPIP2;2* was significantly down-regulated at 6:00 h in both petioles and leaves in response to WS, however, was upregulated at 6:00 h in Grenache petioles.

**FIGURE 7:**
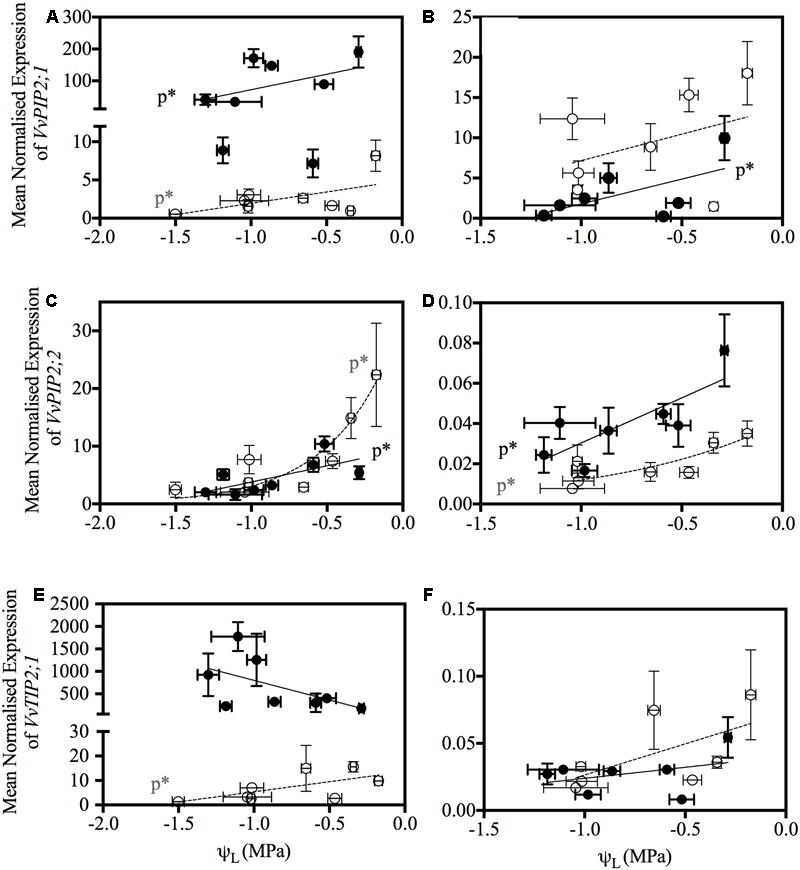
Relationship between aquaporin gene expression and leaf water potential (ψ_L_) in Chardonnay (open gray circles, dotted line) and Grenache (filled black circles, bold line) petioles and leaves. Expression of *VvPIP2;1* in petioles **(A)** and leaves **(B)**, *VvPIP2;2* in petioles **(C)** and leaves **(D)** and *VvTIP2;2* in petioles **(E)** and leaves **(F)**. Only gene expression data that had a significant Pearson correlation with ψ_L_ are shown. Significance is indicated by p^∗^.

*VvTIP2;1* was down-regulated in both Chardonnay and Grenache in response to water-stress, however, this was only significant at 6:00 h in Grenache leaves and 21:00 h in Chardonnay petioles. The significant down-regulation of *VvPIP2;1* and *VvTIP2;1* compared to well-watered plants also correlated with significant decreases in leaf water potential (**Figure 7**).

### Correlation of Grapevine Physiology with Aquaporin Gene Expression

A correlation analysis of the physiological data (time, ψ_L_, ψ_s_, *g*_S_, *E, K*_init_, *K*_Pet_h_, *K*_Pet_LA_, and PLC) with aquaporin gene expression revealed some significant results (**Figure 6**). In Chardonnay ψ_L_ and ψ_S_ positively correlated with time and negatively correlated with *g*_S_, *E* and hydraulic conductivity measurements (*K*_init_, *K*_Pet_h_, and *K*_Pet_LA_) (**Figure 6A**). ψ_L_ and ψ_S_ both positively correlated with PLC. *VvTIP2;1*_petiole_ negatively correlated with PLC in Chardonnay. In Chardonnay, ψ_L_ and ψ_S_ were negatively correlated with aquaporin gene expression of *VvPIP2;1*_petiole_, *VvPIP2;2*_petiole_, *VvTIP2;1*_petiole_, and *VvPIP2;2*_leaf_ (**Figures 6A, 7**). Expression of *VvPIP2;1*_petiole_ and *VvTIP2;1*_petiole_ positively correlated with *g*_S_ and *E*. Hydraulic conductivity measurements (*K*_init_, *K*_Pet_h_, and *K*_Pet_LA_) correlated with time in Chardonnay but not Grenache.

In Grenache, ψ_L_ and ψ_S_ negatively correlated with aquaporin expression of *VvPIP2;1*_petiole_, *VvPIP2;2*_petiole_, and *VvPIP2;2*_leaf_ (**Figure 7**). ψ_S_ negatively correlated with both *g*_S_ and *E* and *VvPIP2;1*_leaf_. *g*_S_, *E*, and *K* parameters did not correlate with ψ_L_ in Grenache (**Figure 6B**). Expression of *VvPIP2;1*_leaf_ positively correlated with the expression of *VvPIP2;1*_petiole_, *VvPIP2;3*_petiole_, *VvPIP2;3*_leaf_, and *VvTIP1;1*_leaf_ (**Figure 6B**). *g*_S_ and *E* correlated with time in Grenache but not Chardonnay.

## Discussion

### Hydraulic Properties Differ between Grapevine Cultivars

Chardonnay vines displayed hydraulic segmentation, with petioles being more susceptible to cavitation than stems (**Figure 1A** and **Table 2**). Previous studies on grapevines have shown the roots to be more vulnerable to cavitation than the shoots and this seems to be common in anisohydric species in order to protect the stem from catastrophic cavitation during drought (Lovisolo and Schubert, 2006; Lovisolo et al., 2008a). Hydraulic segmentation has been reported in woody species including *Juglans regia* L. (Tyree et al., 1993), *Acer saccharum* (Tsuda and Tyree, 1997) with highest cavitation vulnerability for these species in the petioles. Grapevines also display vulnerability segmentation with a number of studies showing stems are more resistant to water-stress induced embolism than petioles (Alsina et al., 2007; Choat et al., 2010; Zufferey et al., 2011; Charrier et al., 2016). Hydraulic vulnerability segmentation in grapevine has been demonstrated in both cv. Syrah and Cabernet Sauvignon using X-ray microcomputed tomography (Charrier et al., 2016; Hochberg et al., 2016). Higher vulnerability in petioles compared to shoots has been proposed to be a form of hydraulic segmentation leading to leaf shedding in response to drought (Tyree et al., 1993). In this study Chardonnay vines maintained midday leaf water potential close to the cavitation threshold (-1.8 MPa for stems) as has been reported for other anisohydric species (McDowell et al., 2008). This is in good agreement with other studies; NMR imaging showed dehydrated Chardonnay vines only suffered significant stem embolism ψ_L_ < -2.0 MPa, and when ψ_L_ was above -1.5 MPa the majority of vessels remained filled (Choat et al., 2010). In Cabernet Sauvignon vines imaged with X-ray microcomputed tomography, embolized vessels increased when ψ_L_< -1.5 MPa and ψ_50Stem_= -1.73 and ψ_50Petiole_= -0.98 MPa. Grenache was more susceptible to water-stress induced cavitation as indicated by the higher ψ_10_ at which cavitation begins to occur, however, showed no evidence of hydraulic segmentation between the stems and petioles (**Figure 1B** and **Table 2**). In this study, under moderate water-stress, Grenache did not show any evidence of developing run-away cavitation as was observed for Chardonnay stems (Supplementary Figure S2). Grenache stems have previously been reported to be more susceptible to the formation of xylem embolism than both Syrah and Chardonnay (Schultz, 2003; Alsina et al., 2007).

It has been proposed that early cavitation events may act as a hydraulic signal for stomatal closure (Salleo et al., 2000) and involves chemical signals such as ABA that may promote embolism repair (Lovisolo et al., 2008a). The early onset of xylem embolism in Grenache stems and petioles may be responsible for triggering midday stomatal closure thus contributing to their near-isohydric behavior (**Figure 2** and **Table 1**). Stomata also respond to transient changes in leaf water potential (Meinzer et al., 2001) that can occur as a result of cavitation, and this may influence the occurrence of stomatal patchiness (During and Loveys, 1996).

Grenache exhibited higher petiole specific hydraulic conductivity than Chardonnay and this is most likely due to the higher frequency of larger xylem vessel diameters (**Figure 5**) (Scholander et al., 1955; Essau, 1965; Lovisolo and Schubert, 1998). The relative measured K_Pet-h_ between cultivars (Chardonnay/Grenache = 0.71) was consistent with the relative *d*_hyd_ (Chardonnay/Grenache = 0.72) and suggests that Grenache are adapted to supply a greater leaf surface area than Chardonnay. This difference was also observed in the rachis xylem comparing Grenache and Shiraz (Scharwies and Tyerman, 2017).

A close relationship between petiole hydraulic properties and vine water status (ψ_L_ and ψ_S_) was observed in Chardonnay vines, but not in Grenache vines (**Figure 3C**). The decline in petiole hydraulic conductivity with increasing water-stress, may be reflective of a decline in the permeability of the water conducting pathway either through the xylem vessels or by way of the cell to cell pathway (via aquaporins). Many studies have examined the contribution of aquaporins in the cell-to-cell pathway of water movement in roots and there is substantial evidence that increases in root hydraulic conductance are correlated with aquaporin expression (Javot and Maurel, 2002; Tyerman et al., 2002; Vandeleur et al., 2009; Chaumont and Tyerman, 2014). Aquaporin’s have also been shown to contribute to leaf hydraulic conductivity in several species and alter in response to specific environmental cues (Prado and Maurel, 2013). Aquaporin’s are highly expressed in xylem parenchyma cells in several species including Arabidopsis (Prado et al., 2013), walnut (Sakr et al., 2003), and maize (Hachez et al., 2008). Expression in xylem parenchyma cells is crucial for radial water movement from xylem vessels and for the refilling of embolized xylem vessels (Secchi and Zwieniecki, 2016). *VvPIP2;1* expression in the petiole of Chardonnay correlated with vine water status, gas exchange and *K*_Pet_LA_ and therefore may play a significant role in regulating petiole and leaf hydraulics in anisohydric cultivars. *VvPIP2;1* also decreased under water deficit in field grown vines (Dayer et al., 2017). In soybean leaves, differential expression of some aquaporin genes correlated with a midday decrease in *K*_Pet_LA_ indicative of a potential role in regulating diurnal fluctuations in leaf water status (Locke and Ort, 2015).

### Aquaporin Genes Are Diurnally Expressed in Grapevine Petioles and Leaves

Both PIP and TIP aquaporins (mRNA and protein) have been shown to be diurnally regulated, with expression generally higher in the day correlating with transpiration. Diurnal and/or circadian changes in aquaporin expression have been observed in maize and Arabidopsis roots (Lopez et al., 2003; Takase et al., 2011; Caldeira et al., 2014) and in the leaves of *Nicotiana excelsior* (Yamada et al., 1997), *Samanea saman* (Moshelion et al., 2002), and maize (Hachez et al., 2008). A diurnal mRNA expression pattern was evident in the petioles and leaves for both Chardonnay and Grenache, however, the response was cultivar, isoform and tissue dependent.

The most predominant diurnal expression pattern was for *VvPIP2;1. VvPIP2;1* expression in both the leaves and petioles of Chardonnay and Grenache was strongly diurnally regulated under well-watered conditions with expression highest predawn and decreasing over the course of the day (**Table 5** and Supplementary Figures S3–S6). A diurnal pattern was still evident even under water-stress conditions, however, in Grenache leaves and petioles expression was decreased compared to well-watered conditions and the peak in expression was at 11:00 h. This may reflect the near-isohydric nature of Grenache, where the transpirational demand tends to be highest in the morning. A strong diurnal regulation of PIP aquaporin’s has previously been reported in the leaves of *Fragaria vesca* (strawberry) under both well-watered conditions and drought conditions (Šurbanovski et al., 2013). The expression of *VvPIP2;1* correlated with ψ_L_ in both Chardonnay and Grenache indicating that expression of this aquaporin isoform responds to leaf water status (**Figure 7**). The high expression predawn in both Chardonnay and Grenache petioles may be consistent with a role in night-time refilling of xylem vessels under well-watered conditions.

*VvPIP2;2*, also demonstrated an apparent diurnal regulation with expression highest at predawn and decreasing during the day in both Chardonnay and Grenache leaves and petioles, with night-time expression increasing to values close to predawn expression in all samples except Grenache leaves. *VvPIP2;2* expression in the leaves and petioles was negatively correlated with plant water status in both Chardonnay and Grenache, indicating that the expression of this gene is finely tuned to both diurnal fluctuations in plant water status and in response to water-stress (**Figure 7**).

In contrast to the *VvPIP2* genes, expression of *VvTIP1;1* increased during the late afternoon in the petioles of Grenache when ψ_L_ was most negative (**Table 3**). This is consistent for a role of TIPs maintaining plant water status during high transpiration by facilitating water movement from the vacuoles. In Grenache leaves, *VvTIP1;1* expression was negatively correlated with stomatal conductance and transpiration (**Figure 6**). Diurnal changes in *TIP* expression have previously been reported in the guard cells of sunflower and are thought to be involved in closing of stomata (Sarda et al., 1997). In Chardonnay petioles, *VvTIP2;1* correlated with both leaf and stem water status, thus the transcriptionally regulation of *VvTIPs* is different in anisohydric/near-isohydric cultivars.

### Aquaporin Gene Expression in Response to Water-Stress

The differences in aquaporin gene expression in response to water-stress in Chardonnay and Grenache is a molecular reflection of the different physiological properties of these two cultivars under water-stress. Changes in aquaporin gene expression in plants has been previously reported in response to abiotic stresses including drought (Yamaguchi-Shinozaki et al., 1992; Yamada et al., 1995; Mariaux et al., 1998; Barrieu et al., 1999; Sarda et al., 1999; Secchi et al., 2007, 2016; Vandeleur et al., 2009; Pou et al., 2013), salt stress (Suga et al., 2002), light (Cochard et al., 2007), cold stress (Li et al., 2000), and diurnal fluctuations (Lopez et al., 2003). The response of aquaporins to water-stress is species and isoform dependent, and in grapevine, also cultivar dependent. Previous studies have demonstrated that grapevine cultivars can either exhibit isohydric or anisohydric responses and this may be linked to aquaporin expression (Vandeleur et al., 2009).

Expression of *VvPIP2;1* and *VvPIP2;2* in Chardonnay petioles, showed a strong, rapid down-regulation in response to water-stress (decrease in ψ_L_) compared with Grenache petioles (**Table 6** and **Figures 7A,B**). Refilling of embolized vessels has been observed in Chardonnay stems under non-transpiring conditions (Brodersen et al., 2010); however, the strong down-regulation of aquaporins under water-stress in this study indicates refilling is unlikely in Chardonnay, at least under transpiring conditions. *VvPIP2;1* has previously been shown to be expressed in VACs of embolized and recovering petioles in Grenache supporting the hypothesis that aquaporins play a major role in xylem refilling (Chitarra et al., 2014). Interestingly, a number of PIP and TIP genes showed increased expression predawn (*VvPIP2;2* was significant) in response to water-stress in Grenache petioles perhaps indicating a potential role in night-time refilling in this cultivar (Secchi et al., 2007). Night-time refilling in Grenache may prevent the onset of run-away cavitation as water-stress increases in severity. VvPIP2;1 and VvPIP2;2 share high homology with the walnut aquaporins, JrPIP2;1 and JrPIP2;2, both postulated to be involved in embolism refilling and expressed in the xylem parenchyma cells of walnut (Sakr et al., 2003), thus these may be a good candidates for modulating refilling in grapevine petioles. In *V. labrusca* L. cv. Concord (fox grape), embolized vessels formed in the stem while the plant was actively transpiring and under considerable water-stress (Holbrook et al., 2001). Refilling of these vessels occurred only when combined with an increase in leaf water potential and a cessation of sap flow. Other authors have proposed that refilling of embolized vessels can occur when transpiration rates are high (McCully et al., 1998; McCully, 1999). Refilling of grapevine vessels has been shown in Chardonnay at a moderate water-stress under non-transpiring conditions and is believed to be dependent on water movement from the living xylem parenchyma cells into the xylem vessels (Brodersen et al., 2010). Given the physiological and molecular differences observed between Chardonnay and Grenache it is possible that different refilling strategies exist between these cultivars.

In leaves, the aquaporin expression profile differed to petioles, with Grenache exhibiting greater down-regulation of aquaporin genes in response to water-stress than Chardonnay. In Chardonnay leaves, the expression of aquaporin genes was isoform specific, with *VvPIP2;2* and *VvTIP2;1* decreasing expression relative to well-watered (similar to Grenache) yet other genes only showed small insignificant increases/decreases in expression. Interestingly, *VvPIP1;1* that is thought to be involved in regulation of other aquaporins, was upregulated in petioles and leaves in response to water-stress in both cultivars. This is most likely a reflection of the different tissue types, with petioles primarily behaving as water conduits to the photosynthetic machinery in the leaves. Increased aquaporin expression could be linked to photosynthesis (Hachez et al., 2008) and/or embolism repair (Holbrook et al., 2001). The regulation of aquaporin gene expression in Grenache leaves appears to be related to the near-isohydric behavior and conservative water use strategy, whereas the response of aquaporin’s in Chardonnay leaves is less dramatic, and may reflect the anisohydric behavior of this cultivar.

The overexpression of *SoTIP2;2* in tomato has been suggested to be involved in regulating anisohydric behavior in response to drought stress by maintaining vacuolar water permeability and thus osmotic buffering in response to abiotic stress conditions (Sade et al., 2009). Overexpression of *VvPIP2;4N* in the anisohydric cultivar, Brachetto, resulted in greater leaf capacitance compared to wildtype, indicating that aquaporins’ may be involved in the control of hydraulic capacitance in grapevine (Vitali et al., 2016). *VvTIP2;1* expression in Chardonnay petioles correlates with both gas exchange and water status and thus may help regulate vacuolar water permeability in response to water-stress conditions in anisohydric grapevine cultivars, perhaps through control of hydraulic capacitance. A link between aquaporins’ and hydraulic capacitance needs to be further evaluated in iso/anisohydric cultivars.

## Conclusion

In summary, the data show that there were two contrasting responses in petiole hydraulics and aquaporin expression between the near-isohydric cultivar Grenache and anisohydric cultivar Chardonnay. We have shown that Grenache (near-isohydric variety) was more susceptible to the onset of xylem embolism in both the petioles and stems than Chardonnay (anisohydric), most likely linked to larger xylem vessels. It is apparent that Grenache employs a molecular and physiological strategy to conserve cellular water balance. To further understand the role of aquaporin’s in isohydric/anisohydric response to water-stress, further investigation into both the transcriptional and post-translational regulation of aquaporin’s in specific cell types (i.e., xylem parenchyma cells) needs to be investigated. The different water use strategy of these two cultivars, Chardonnay and Grenache needs to be accounted for in irrigation management. Furthermore, the petiole and leaf signature of expressed aquaporin’s may be used in combination with other petiole assessments in screening different genotypes for differences in isohydric/anisohydric behavior.

## Author Contributions

MS, BK, and ST conceived and designed the experiments. MS, RV, and ST conducted the experiments and analyzed the data. MS and ST wrote the manuscript and all authors contributed to editing the final manuscript.

## Conflict of Interest Statement

The authors declare that the research was conducted in the absence of any commercial or financial relationships that could be construed as a potential conflict of interest.

## Footnotes

1 http://frodo.wi.mit.edu/primer3/

2 http://www.rstudio.com/
