# Supplementary material for: A Comparison of Petiole Hydraulics and Aquaporin Expression in an Anisohydric and Isohydric Cultivar of Grapevine in Response to Water-Stress Induced Cavitation
Source: Front Plant Sci. 2017 Nov 7;8:1893. doi: 10.3389/fpls.2017.01893 (PMC5681967; doi:10.3389/fpls.2017.01893)
Supplement: Supplementary file 1 [file Data_Sheet_1.DOCX]

**S****upplementary Material**

**A Comparison of Petiole Hydraulics and Aquaporin Expression in an Anisohydric and Isohydric Cultivar of Grapevine in Response to Water-Stress Induced Cavitation**

**Megan C. Shelden^*^, Rebecca K. Vandeleur, Brent N. Kaiser, Stephen D. Tyerman**

***Correspondence:** Dr. Megan Shelden**,** [megan.shelden@adelaide.edu.au](mailto:megan.shelden@adelaide.edu.au)

**Supplementary Figures and Tables**

**Supplementary Table 1**: Accession numbers of aquaporin genes and gene specific primer sequences for quantitative-PCR. The length of amplicon for each primer set is also shown.

| Gene | Accession No. | Primer Sequence (5’- 3’) | Length(bp) |
| --- | --- | --- | --- |
| *VvPIP1;1* | AF141643 | F - TGGTGCGGGTGTAGTGAAGG  R AGACAGTGTAGACAAGGACGAAGG | 143 |
| *VvPIP2;1* | AY823263 | F - CAGGAGCACCACTCATGTATG  R TCATGCCCTCATACATATCAATAAC | 152 |
| *VvPIP2;2* | EF364436 | F - AAAGTTTGGGACGACCAGTG  R - TTTTTAGTTGGTGGGGTTGC | 141 |
| *VvPIP2;3* | EF364437 | F - GCCATTGCAGCATTCTATCA  R - TCCTACAGGGCCACAAATTC | 199 |
| *VvTIP1;1* | AY839872 | F - CATTGCCGCCATCATCTAC  R - AGAAATCTCAACCCCACCAG | 156 |
| *VvTIP2;1* | EF364439 | F - GGAGGAAGAGCAAGTTGTGC  R GCACATCACCAACCTCATTC | 157 |
| *Vl x Vv eEF1γ* | AF176496 | F - CGGGCAAGAGATACCTCAAT  R - AGAGCCTCTCCCTCAAAAGG | 147 |
| *VvActin7* | XM002282480 | F - GCCTCCGATTCTCTCTGCTCTC  R - TCACCATTCCAGTTCCATTGTCAC | 110 |
| *VvUbq60s* |  | F - GTGCTGTCAACTGCAGGAAA  R - GTAGCCATGGCACATCCAA | 140 |

**Supplementary Figure S1:** Correlation of leaf water potential of Chardonnay measured with the pressure chamber and leaf psychrometers. Leaf water potential was measured continuously on pot grown vines with water withheld for 6 days. Leaf psychrometers were attached to mature leaves and leaf water potential recorded every 15 min over the experimental period. Pressure chamber measurements were taken once a day at 13:00 h on one leaf from each vine. A linear correlation (bold line) found no significant difference when compared to a slope of 1, with 95% confidence interval (dotted line). Data represents the mean ± SEM (n=3).

**Supplementary Figure** **S2**: Representative time course of leaf water potential (ψ_L_) and cavitation (UAE) over a water-stress treatment in pot grown (a) Chardonnay and (b) Grenache grapevines. Shown is the mean leaf water potential (black line) for one vine recorded continuously with leaf psychrometers (n = 4) every 15 min over the water-stress period (7 days). Cavitation was detected simultaneously in the petiole (blue line) and stem (red line) by measuring the UAEs.

**Supplementary Figure** **S3:** Percent Loss of Conductance (PLC) measured over a diurnal period in the petioles of WW (closed circles, bold line) and WS (open circles, dotted line) in Chardonnay and Grenache vines. Superscript letters indicate significant between treatment at each time point (p < 0.05).

**Supplementary Figure S4**: Aquaporin transcript abundance in the petioles of well-watered (black bars) and water-stressed (white bars) Chardonnay vines. Petioles were harvested over a diurnal period from WW and WS vines. Quantitative real time PCR was used to determine the mean normalised expression ± SE (n = 3- 4) for the following genes **(A)** *VvPIP1;1* **(B)** *VvPIP2;1* (**C)** *VvPIP2;2* **(D)** *VvPIP2;3* **(E)** *VvTIP1;1* and **(F)** *VvTIP2;1.* Significance in gene expression between WW and WS at each timepoint is indicated by * (p < 0.05). * indicates significance for each timepoint compared to 6:00 h for WW.

**Supplementary Figure S5:** Aquaporin transcript abundance in the petioles of well-watered (black bars) and water-stressed (white bars) Grenache vines. Petioles were harvested over a diurnal period from WW and WS vines. Quantitative real time PCR was used to determine the mean normalised expression ± SE (n = 3- 4) for the following genes **(A**) *VvPIP1;1* **(B)** *VvPIP2;1* **(C)** *VvPIP2;2* **(D)** *VvPIP2;3* **(E)** *VvTIP1;1* and **(F)** *VvTIP2;1.* Significance in gene expression between WW and WS at each timepoint is indicated by a bar and ** (p < 0.05). * indicates significance for each timepoint compared to 6:00 h for WW.

**Supplementary Figure S6:** Aquaporin transcript abundance in the leaves of well-watered (black bars) and water-stressed (white bars) Chardonnay vines. Petioles were harvested over a diurnal period from WW and WS vines. Quantitative real time PCR was used to determine the mean normalised expression ± SE (n = 3- 4) for the following genes **(A)** *VvPIP1;1* **(B)** *VvPIP2;1* (**C)** *VvPIP2;2* **(D)** *VvPIP2;3* **(E)** *VvTIP1;1* and **(F)** *VvTIP2;1.* Significance in gene expression between WW and WS at each timepoint is indicated by * (p < 0.05). * indicates significance for each timepoint compared to 6:00 h for WW.

**Supplementary Figure S7:** Aquaporin transcript abundance in the leaves of well-watered (black bars) and water-stressed (white bars) Grenache vines. Leaves were harvested over a diurnal period from WW and WS vines. Quantitative real time PCR was used to determine the mean normalised expression ± SE (n = 3- 4) for the following genes **(A)** *VvPIP1;1* **(B)** *VvPIP2;1* **(C)** *VvPIP2;2* **(D)** *VvPIP2;3* **(E)** *VvTIP1;1* and **(F)** *VvTIP2;1.* Significance in gene expression between WW and WS at each timepoint is indicated by a bar and ** (p < 0.05). * indicates significance for each timepoint compared to 6:00 h for WW.
